# The Locus Coeruleus–Norepinephrine System Mediates Empathy for Pain through Selective Up-Regulation of P2X3 Receptor in Dorsal Root Ganglia in Rats

**DOI:** 10.3389/fncir.2017.00066

**Published:** 2017-09-20

**Authors:** Yun-Fei Lü, Yan Yang, Chun-Li Li, Yan Wang, Zhen Li, Jun Chen

**Affiliations:** ^1^Institute for Biomedical Sciences of Pain, Tangdu Hospital, The Fourth Military Medical University Xi’an, China; ^2^Key Laboratory of Brain Stress and Behavior, PLA Xi’an, China; ^3^Anesthesia and Operation Center, 302 Military Hospital Beijing, China; ^4^Beijing Institute for Brain Disorders Beijing, China

**Keywords:** empathy, locus coeruleus-norepinephrine system, sympatho-adrenomedullary axis, hypothalamic-pituitary-adrenocortical axis, adrenoceptor, P2X3 receptor, mechanical hyperalgesia, pain

## Abstract

Empathy for pain (vicariously felt pain), an ability to feel, recognize, understand and share the painful emotions of others, has been gradually accepted to be a common identity in both humans and rodents, however, the underlying neural and molecular mechanisms are largely unknown. Recently, we have developed a rat model of empathy for pain in which pain can be transferred from a cagemate demonstrator (CD) in pain to a naïve cagemate observer (CO) after 30 min dyadic priming social interaction. The naïve CO rats display both mechanical pain hypersensitivity (hyperalgesia) and enhanced spinal nociception. Chemical lesions of bilateral medial prefrontal cortex (mPFC) abolish the empathic pain response completely, suggesting existence of a top-down facilitation system in production of empathy for pain. However, the social transfer of pain was not observed in non-cagemate observer (NCO) after dyadic social interaction with a non-cagemate demonstrator (NCD) in pain. Here we showed that dyadic social interaction with a painful CD resulted in elevation of circulating norepinephrine (NE) and increased neuronal activity in the locus coeruleus (LC) in the CO rats. Meanwhile, CO rats also had over-expression of P2X3, but not TRPV1, in the dorsal root ganglia (DRG). Chemical lesion of the LC-NE neurons by systemic DSP-4 and pharmacological inhibition of central synaptic release of NE by clonidine completely abolished increase in circulating NE and P2X3 receptor expression, as well as the sympathetically-maintained development of empathic mechanical hyperalgesia. However, in the NCO rats, neither the LC-NE neuronal activity nor the P2X3 receptor expression was altered after dyadic social interaction with a painful NCD although the circulating corticosterone and NE were elevated. Finally, in the periphery, both P2X3 receptor and α1 adrenergic receptor were found to be involved in the development of empathic mechanical hyperalgesia. Taken together with our previous results, empathy for pain observed in the CO rats is likely to be mediated by activation of the top-down mPFC-LC/NE-sympathoadrenomedullary (SAM) system that further up-regulates P2X3 receptors in the periphery, however, social stress observed in the NCO rats is mediated by activation of both hypothalamic-pituitary-adrenocortical axis and SAM axis.

## Introduction

Pain can be perceived not only directly by self experience but also vicariously through social interaction and communication (Langford and de C Williams, 2014; Martin et al., 2014; Mogil, 2015; Williams and Craig, 2016; Chen, 2017). The vicariously felt pain of others is believed to be dependent upon empathy, an ability to feel, recognize, understand and share the painful emotional state of others (Preston and de Waal, 2002; de Waal, 2008; Bernhardt and Singer, 2012; Decety et al., 2016; Chen, 2017). It has been demonstrated that both directly felt pain and vicariously felt pain are mediated by a common neural network mainly involving the anterior cingulate cortex (ACC) and anterior insular cortex (Rainville et al., 1997; Singer et al., 2004; Lamm et al., 2011). Empathy for pain has been shown to be able to change the way an individual feels pain (social modulation of pain), leading to pain hypersensitivity (hyperalgesia or allodynia) in humans (Goubert et al., 2005; Loggia et al., 2008; Godinho et al., 2012).

Although traditionally empathy was believed to be a unique identity of human beings, it has been recently accepted to be a common sentience of both humans and animals (for reviews, see Panksepp and Lahvis, 2011; Gonzalez-Liencres et al., 2013; Panksepp and Panksepp, 2013; Martin et al., 2014; Mogil, 2015; Keum and Shin, 2016; Sivaselvachandran et al., 2016; Chen, 2017; Meyza et al., 2017). In rodents, empathy for pain has been demonstrated to exist at least in two well-designed studies: one is referred to as emotional contagious pain transferred through on-site social interaction between dyadic familiar mice (both of them are in pain; Langford et al., 2006); the other is referred to as empathy for pain produced through priming social interaction between dyadic familiar rats (one in pain as a demonstrator and the other without pain as a naive observer; Li et al., 2014). It is interesting to find that establishment of familiarity among conspecifics by co-housing them for more than 2 weeks (also referred to as cagemate) is necessary for production of empathy for pain in both mice and rats (Martin et al., 2014; Mogil, 2015; Chen, 2017). Specifically in the rat model, it was shown that after priming social interaction with a cagemate in pain produced by subcutaneous (s.c.) injection of bee venom (BV), the naïve observer rat showed mechanical pain hypersensitivity reflected by lowered paw withdrawal mechanical threshold (PWMT) in both sides of the limbs, however, thermal pain sensitivity assessed by paw withdrawal thermal latency (PWTL) was not altered (Li et al., 2014). The empathic mechanical pain hypersensitivity (or hyperalgesia) identified in naïve observer rats has been further demonstrated to be mediated by top-down facilitation from medial prefrontal cortex (mPFC) to the spinal dorsal horn (Li et al., 2014). However, so far the underlying molecular and neural mechanisms of empathy for pain in rodents remain largely unclear. It is known that cognition and emotion can modulate pain at both spinal and supraspinal levels (Bantick et al., 2002; Villemure et al., 2003; Rhudy et al., 2005, 2008; Dunckley et al., 2007; Williams and Rhudy, 2007; Roy et al., 2009; Villemure and Bushnell, 2009; Bushnell et al., 2013; Liu and Chen, 2014), however, whether they can modulate pain at the peripheral level is largely unknown. It is interesting to note that neural responses of primary olfactory neurons to threat-predictive odor stimuli could be enhanced by fear learning (Kass et al., 2013), inspiring our hypothetical account of top-down regulation of primary nociceptors by empathic responses from the brain.

The hypothalamo-pituitary-adrenocortical (HPA) axis and the sympatho-adrenomedullary (SAM) axis are two main neuroendocrine systems that maintain adaptive ability in response to various harmful stressors (pain, fear and catastrophe; Chrousos, 2009; Ulrich-Lai and Herman, 2009). Pain sensitivity can be bidirectionally modulated by stress—a hyperfunctional state of the HPA axis, resulting in either stress-induced analgesia (Butler and Finn, 2009) or stress-induced hyperalgesia (Jørum, 1988; Imbe et al., 2006; Jennings et al., 2014; Olango and Finn, 2014). However, in social activities of both humans and animals, it is likely that the functional state of the HPA axis can be enhanced by social stress which strongly prevents empathy for pain or distress from occurring (Chen, 2017). For example, it has been demonstrated that emotional contagion of pain failed to occur after social interaction between strangers in both humans and mice due to socially stress-induced rise in circulating level of corticosterone (CORT; Martin et al., 2015). However, it could be elicited between strangers by pharmacological inhibition of the synthesis of CORT or antagonisms against both glucocorticoid (GC) and mineralocorticoid (MC) receptors (Martin et al., 2015). On the other hand, restraint-induced stress for more than 30 min was enough to block emotional contagion of pain between familiar mice (Martin et al., 2015). However, whether the HPA axis plays a role in the development of empathy for pain in rats requires to be further addressed.

The locus coeruleus (LC), which is the main source of norepinephrine (NE, or noradrenaline) in the whole nervous system, is known to play very important roles in cognitive and emotional processes (Aston-Jones et al., 1999; Berridge and Waterhouse, 2003; Bouret and Sara, 2005; Sara, 2009; Sara and Bouret, 2012). The functional state of the SAM axis can be largely controlled by the LC-NE system (Berridge and Waterhouse, 2003). The LC-NE system can also modulate pain through both supraspinal and spinal actions (Jørum, 1988; Proudfit, 1988; Jones, 1991; Pertovaara, 2006). However, whether the LC-NE system has a role in the development of empathy for pain is totally unknown. The P2X3 receptor, a subtype of ligand-gated P2X purinoceptor of adenosine triphosphate (ATP), has been proposed to be a nociceptor molecule identified in non-peptidergic neurons of the dorsal root ganglion (DRG; Burnstock, 2000). P2X3 receptor has been shown to be upregulated in pathological pain states (Novakovic et al., 1999), and selective P2X3 receptor antagonism can alleviate both mechanical and thermal hyperalgesia in different experimental pain models (Burnstock, 2000; Jarvis et al., 2002; McGaraughty et al., 2003; Lu et al., 2008). It is intriguing to note that P2X3 receptor-mediated nociceptive response can be enhanced by noradrenergic pathways in rat DRG neurons (Maruo et al., 2006; Meisner et al., 2007). Moreover, NE is able to exert direct effects on P2X3 receptors, whereby incubation of cultured DRG neurons with NE can upregulate the expression of P2X3 receptors (Tan et al., 2011). Based upon the above evidence, a question is raised to ask whether the empathic mechanical hyperalgesia is mediated by activation of peripheral P2X3 receptors through top-down facilitation by the mPFC—LC-NE system—spinal circuitry. TRPV1, transient receptor potential vanilloid subtype 1, is known to be a thermal nociceptor molecule that is localized in both the peptidergic and non-peptidergic DRG neurons (Caterina et al., 1997; Julius, 2013). Whether peripheral TRPV1 is involved in the development of the empathic mechanical hyperalgesia is unknown and also requires to be examined.

Therefore the present study was designed to examine: (1) functional states of the HPA axis and the SAM axis in rats after priming social interaction with a naïve cagemate, a cagemate in pain and a non-cagemate in pain; (2) expression level of nociceptor proteins (P2X3 and TRPV1) in the DRG obtained from rats after priming social interaction with a naïve cagemate, a cagemate in pain and a non-cagemate in pain; (3) roles of the central LC-NE system in regulation of nociceptor protein P2X3 and mediation of empathy for pain in rats; and (4) roles of peripheral noradrenergic receptor subtypes in mediation of empathy for pain in rats.

## Materials and Methods

### Animals

Only male Sprague-Dawley rats (180–250 g, 8–10 weeks old), purchased from the Laboratory Animal Center of the Fourth Military Medical University (FMMU), Xi’an, Shaanxi Province, China, were used in the present study. After arrival at the SPF animal facility at our lab, the animals were housed under standard conditions (12 h dark/light cycles, temperature 22–26°C, air humidity 40%–60%) in groups of 4–6 with free access to food and water. The present experimental procedures were approved by the Institutional Animal Care and Use Committee of FMMU (reference No. 20150202). All animal experiments complied with the ARRIVE guidelines (Kilkenny et al., 2010) and carried out in accordance with the U.K. Animals (Scientific Procedures) Act, 1986 and associated guidelines, EU Directive 2010/63/EU for animal experiments, and the National Institutes of Health guide for the care and use of Laboratory animals (NIH Publications No. 8023, revised 1978). The ethical guidelines for investigations of experimental pain in conscious animals of the International Association for the Study of Pain were also critically followed (Zimmermann, 1983). Every effort was made to minimize the number of animals used and their suffering. In the present study, cagemate was referred to as rats drawn from the same cage, where they had been co-housed for more than 2 weeks before initiation of the experiment, and were not necessarily siblings. The behavioral experiments were mainly carried out by male experimenters and the experimenters were blind to the treatment of rats.

### Experimental Design

Rats were randomly assigned to three groups as previously reported (Li et al., 2014): (1) cagemate control (CC), a pair of cagemate rats allowed to interact with each other in a transparent plastic box (20 × 20 × 25 cm) for 30 min; (2) naïve cagemate observer (CO), a rat allowed to interact with a cagemate (demonstrator) in pain for 30 min. The pain model was produced by s.c. injection of BV (0.2 mg/50 μl, Sigma, USA) into the plantar surface of a hindpaw of the demonstrator rat who immediately displayed a period of 1 h robust flinching, licking and lifting of the injected paw after being placed back to the transparent plastic box (for method see Chen et al., 1999; Chen and Lariviere, 2010); and (3) naïve non-cagemate observer (NCO), a rat allowed to interact with a non-cagemate in pain induced by BV injection.

In the first part of the study, the rats for CC, CO and NCO groups were subjected to four experiments: (1) time course behavioral assessment of empathy for pain; (2) enzyme-linked immunosorbent assay (ELISA) of circulating concentrations of NE and CORT after 10 min and 30 min priming dyadic social interaction; (3) Western blot testing for expression of nociceptor molecules (P2X3 and TRPV1) in the lumber DRG after 30 min priming dyadic social interaction; (4) neuronal activity in the LC examined by immunohistochemical staining of c-Fos protein, a biomarker of neural activation, after 30 min priming dyadic social interaction.

According to the results of the above experiments, in the second part of the study; (5) roles of the central LC—NE system—SAM axis in regulation of DRG nociceptor molecule P2X3 and mediation of empathy for pain behaviors were studied in the CC and CO rats only; (6) the expression level of P2X3 receptor was measured *in vitro* using Western blot following incubation of L4-L5 DRGs with NE; and (7) modulatory roles of the sympatho-postganglionic NE system and peripheral noradrenergic receptor subtypes in mediation of empathic mechanical pain hypersensitivity were studied in the CO rats.

### Quantitative Measurement of PWMT

Rats were placed in a transparent plastic box (20 × 20 × 25 cm) with metal mesh floor and a series of von Frey filaments with different bending forces (7.84, 19.6, 39.2, 58.8, 78.4, 98.0, 117.6, 137.2, 156.8, 176.4, 196.0, 245.0, 294.0, 441.0 and 588.0 mN) was applied to the center of the bilateral hindpaw with 10-s block and 10 repetitions were done for each side. The forces being able to induce more than five paw withdraw reflexes were considered as the value of PWMT (for details see Chen et al., 1999).

### Neurotoxic Lesion of NE Neurons in the LC

To investigate the role of the central LC noradrenergic system in the development of empathic mechanical pain hypersensitivity, i.p. administration of a single dose (50 mg/kg) of N-(2-chloroethyl)-N-ethyl-2-bromobenzylamine hydrochloride (DSP-4; Sigma, USA), a neurotoxin that can selectively eliminate noradrenergic projections from the LC noradrenergic neurons (Jaim-Etcheverry and Zieher, 1980; Ross and Stenfors, 2015), was conducted. After 15 days of DSP-4 treatment, number of tyrosine hydroxylase (TH, rate-limiting enzyme of catecholamine syntheses) positive neurons in the LC (A6) and the substantia nigra (SN), levels of serum NE, DRG P2X3 expression, and changes in PWMT were measured, respectively, as described above. Noradrenergic neurons were labeled using an antibody against TH.

### Pharmacological Inhibition of Synaptic Release of NE from the LC Neurons

To explore the role of intrinsic noradrenergic neuronal NE in the development of empathic mechanical pain hypersensitivity, intraperitoneal (i.p.) administration of clonidine (10 μg/kg, Sigma, USA), an α2-adrenoceptor agonist and inhibitor of the synaptic release of NE (Berridge and Waterhouse, 2003), was performed in the CO rats 15 min prior to the dyadic priming social interaction. Soon after the dyadic priming social interaction, concentrations of serum NE were measured using ELISA, the expression levels of P2X3 receptors from L4-L5 DRGs were measured using Western blot and bilateral changes in PWMT were measured using von Frey filaments in a group of CO rats, respectively.

### Chemical Sympathectomy

Chemical sympathectomy was performed by s.c. administration of guanethidine (50 mg/kg, each day for 3 days) in the CO rats 3 days prior to dyadic social interaction (Chen et al., 2010) so as to see whether sympathetic innervations have a role in the development of empathic mechanical pain hypersensitivity.

### Western Blot

The DRG neurons from L4 and L5 were collected from rats under anesthesia with chloral hydrate (0.36 g/kg, i.p.) within 5 min after the dyadic social interaction. The total proteins were extracted with RIPA lysis buffer and were then quantified with BCA™ protein assay kit (Thermo Scientific, USA). Samples were heated for 10 min at 95°C. Same amounts of proteins were subjected to electrophoresis and transferred to nitrocellulose membranes (Bio-Rad, USA) and incubated with 5% non-fat milk dissolved in 0.01 M PBS with 0.05% Tween 20 (PBST) for 3 h; then membranes were incubated with primary antibody overnight at 4°C. Following several washes in PBST, membranes were incubated with horseradish peroxidase (HRP)-conjugated secondary antibodies for 3 h. Blots were developed with ChemiGlow West chemiluminescent substrate kit and signals were acquired with FluorChem FC II (Alpha Innotech Corp.). As P2X3 receptor and actin have similar molecular weights, the membranes were washed in stripping buffer after detection of P2X3, and then reused for detection of actin on the same membranes. Relative intensities of targeted bands were analyzed with AlphaImager EP Analysis Software (Cell Biosciences, Inc.). Primary antibodies used in the present study were rabbit anti-P2X3 (1:400, Millipore, USA), mouse anti-TRPV1 (1:200, Alomone, Israel), mouse anti-actin (1:2000, Sigma, USA). The following secondary antibodies were used: Goat anti-rabbit HRP and goat anti-mouse HRP (1:2000, ZSGB-Bio, China).

### ELISA

Blood samples were collected transcardially under anesthesia with chloral hydrate (0.36 g/kg, i.p.) from rats 5 min after the dyadic social interaction, and centrifuged (2000 rpm, 15 min) at room temperature, and the serum was collected. The concentrations of NE were measured with an ELISA kit (Elabscience, China) according to the manufacturer’s instructions.

### DRG Culture

Under anesthesia with chloral hydrate (0.36 g/kg, i.p.), DRGs from L4 and L5 were collected and placed in artificial cerebral spinal fluid (ACSF). After removing the connective tissue, DRGs were digested in a solution of 0.4 mg/ml trypsin and 1.0 mg/ml type-A-collagenase (Sigma, USA) for 40 min at 37°C. Then the intact DRGs were incubated in 100 nM NE (diluted in ACSF) or ASCF, which were oxygenated with 95% O_2_ and 5% CO_2_, at room temperature for 5 h. For method see a previous report (Tan et al., 2011).

### Immunohistochemistry

The CC and CO rats were perfused transcardially with 0.9% physiological saline followed by 4% paraformaldehyde under anesthesia with chloral hydrate (0.36 g/kg, i.p.). The whole brain was removed and post-fixed in 4% paraformaldehyde for 4 h and then subjected to 30% sucrose solution in 0.01 M phosphate-buffered saline (PBS) for cryoprotection, until it sunk to the bottom. Coronal brain sections were cut on a freezing microtome (Leica, Germany). Brain stem sections containing the LC were collected and washed in 0.01 M PBS, followed by 10 min incubation in 3% hydrogen peroxide, and were then incubated in 1% bovine serum and 0.5% Triton-100 in 0.01 M PBS for 1 h. Brain sections were incubated overnight at 4°C with rabbit anti c-Fos polycolonal antibody (1:200, Cell Signaling). Then brain sections were washed in 0.01 M PBS for three times and subjected to incubation of goat-anti-rabbit biotinylated secondary antibody (1:200, Geneshare, China) for 3 h, followed by three washes in 0.01 M PBS and then incubated with avidin-biotin complex (1:200, Sigma, USA) for 3 h. After three washes in PBS, the immunostaining reaction was developed with an ABC kit (ZSGB-Bio, China). The reaction was stopped by washes in PBS and the brain sections were mounted on slides and then coverslipped. The number of c-Fos positive cells in the LC of the CC and CO rats were then quantified. The neurotoxic effects of DSP-4 on LC were determined through counting the number of TH-positive neurons in the LC. Rats that received saline vehicle or DSP-4 injection were subjected to the above described procedures, the brain sections that containing the LC and SN were stained with anti-TH antibody (1:500, Abcam, USA).

### Peripheral Administration of Drugs

Whether A317491 (0.2 mg/10 μl, Sigma, USA), a selective P2X3 receptor antagonist, has blocking effect on the development of empathic mechanical pain hypersensitivity in the CO rats was examined by s.c. injection of the drug into the plantar surface of one hindpaw randomly (left or right) 15 min prior to the dyadic social interactions.

To determine whether peripheral postganglionic sympathetic innervations exists and if exists, which adrenergic receptor subtypes are involved in the development of empathic mechanical pain hypersensitivity, s.c. administration of prazosin (40 μg/25 μl), a selective α1 adrenergic receptor antagonist, yohimbine (30 μg/25 μl), a potent α2 adrenergic receptor antagonist, and propranolol (5 μg/25 μl), a β adrenergic receptor antagonist, was performed, respectively, in the CO rats 15 min prior to the dyadic priming social interaction. The dose selection of the pharmacological agents was based on, but slightly different from, the following studies: A317491 (Hsieh et al., 2012), clonidine (Jørum, 1988), DSP-4 (Ross and Stenfors, 2015), guanethidine (Chen et al., 2010), prazosin (Lee et al., 2000), yohimbine (Silva et al., 2015) and propranolol (Wang et al., 2013). The CC rats and vehicles (0.9% physiological saline) of the above drugs served as controls.

### Statistical Analysis

The artwork was created by GraphPad Prism 5.0 (GraphPad software Inc., La Jolla, CA, USA). All data are expressed as mean ± SEM. Two-way analysis of variance (ANOVA) repeated measures, Student’s *t* test (two independent samples, paired-samples) and one-way ANOVA (followed by *post hoc* Fisher’s LSD—least significance difference) were used to analyze mean differences. *P* < 0.05 was considered to be of statistical significance.

## Results

### Temporal Effects of Dyadic Social Interaction on Mechanical Pain Sensitivity and Functional State of HPA and SAM Axis

**Figure 1:**
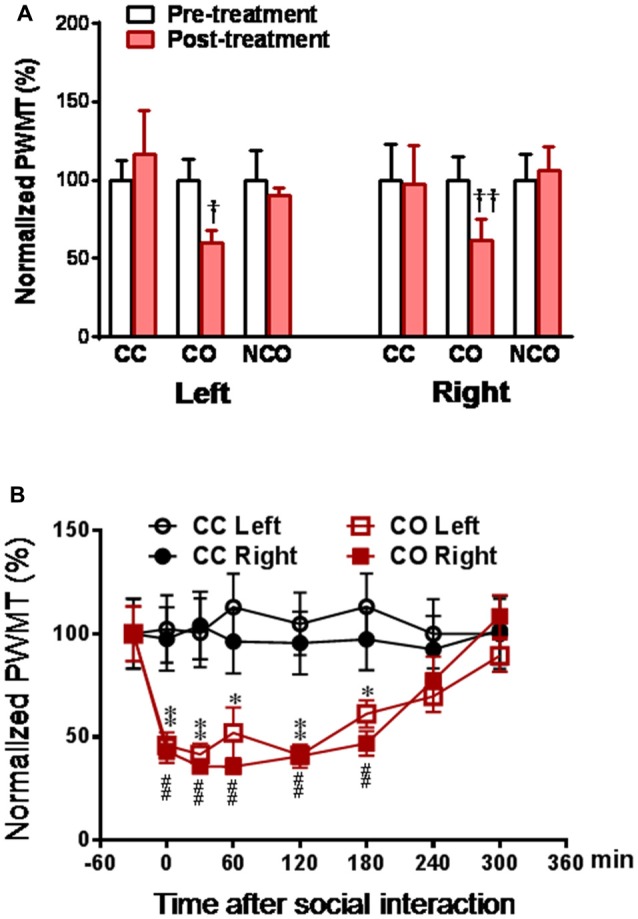
Social interaction with a cagemate in pain induces mechanical pain hypersensitivity in the cagemate observer (CO) rats.** (A)** showing normalized paw withdrawal mechanical threshold (PWMT) in cagemate control (CC), CO and non-cagemate observer (NCO) rats (*n* = 6 per group) following 30 min social interaction. Following measurements of PWMT, the lumbar dorsal root ganglia (DRGs) of CC, CO and NCO rats were removed immediately, and then subjected to molecular and biochemical experiments as shown in Figures 3A–C. **(B)** showing the time course of normalized PWMT in the CC and CO rats (*n* = 10 per group). CC, cagemate control; CO, cagemate observer; NCO, non-cagemate observer, PWMT, paw withdraw mechanical threshold. ^†^*P* < 0.05, ^††^*P* < 0.01 or *P < 0.05, **P < 0.01, or ^##^*P* < 0.01, CO vs. CC at each side. Data are expressed as mean ± SEM.

As previously reported (Li et al., 2014), following 30 min dyadic social interaction with a conspecific in pain, only familiar observer (CO) rat showed bilateral reductions in PWMT (Figure 1A), suggesting selective occurrence of empathic mechanical pain hypersensitivity (hyperalgesia) in familiar conspecific witness. Relative to the CC rats, the time course observation showed that the reduction in PWMT in the CO ones could be identified immediately after dyadic social interaction with a cagemate in pain (Figure 1B). This empathic mechanical hyperalgesia remained less changed during 0–180 min and gradually recovered to the baseline level or the CC control level by 300 min (Figure 1B).

**Figure 2:**
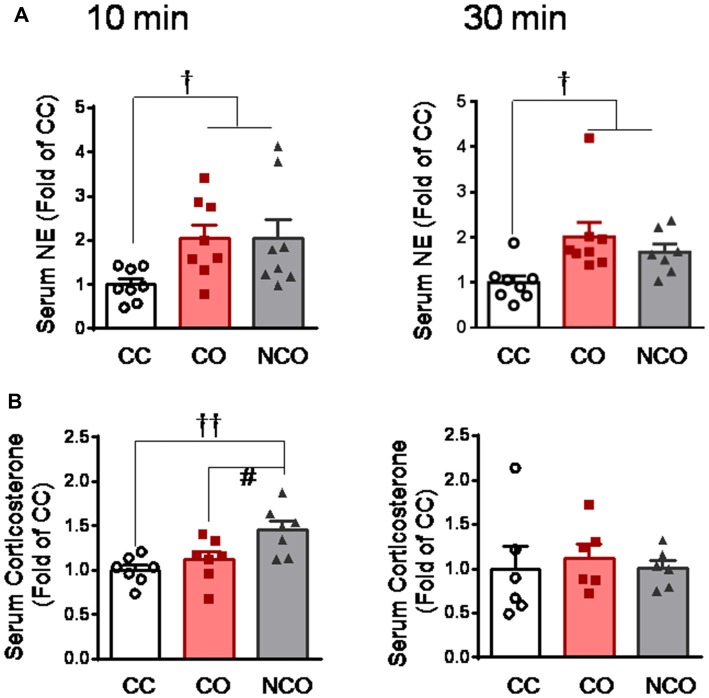
Serum circulating NE and corticosterone levels measured in the CC, CO and NCO rats after 10 min and 30 min dyadic social interaction. **(A)** showing serum NE levels in the CC, CO and NCO rats after 10 min and 30 min social interaction. **(B)** showing serum corticosterone levels in the CC, CO and NCO rats after 10 min and 30 min social interaction. Data are normalized to that of CC rats. CC, cagemate control; CO, cagemate observer; NCO, non-cagemate observer, NE, norepinephrine. ^†^*P* < 0.05, ^††^*P* < 0.01 vs. CC; ^#^*P* < 0.05 NCO vs. CO. Data are expressed as mean ± SEM.

To explore the functional state of the SAM axis and HPA axis after dyadic social interaction with a conspecific in pain, we measured concentration of serum NE and CORT, respectively, by ELISA assay. As a consequence, the level of circulating NE was significantly increased in both the CO and NCO rats at 10 min and 30 min after social interaction with a conspecific in pain, relative to the CC control (CO or NCO vs. CC, *P* < 0.05 for both 10 min and 30 min, *n* = 7–8 for each group; Figure 2A), however, the level of circulating CORT was only shortly increased in the NCO rats at 10 min after social interaction with a conspecific in pain (NCO vs. CC or CO, *P* < 0.01 or *P* < 0.05, *n* = 6–7 for each group) while that in the CO rats was not significantly changed at either 10 min and 30 min after witnessing a familiar in pain, relative to the CC (CO vs. CC, *P* > 0.05, *n* = 6–7 for both 10 min and 30 min; Figure 2B). These results suggest that the SAM axis rather than the HPA axis can be stably activated in rats witnessing a conspecific in pain regardless of either familiar or unfamiliar.

### Influence of Dyadic Social Interaction with a Conspecific in Pain on the Expression Level of P2X3 and TRPV1 in the DRG

**Figure 3:**
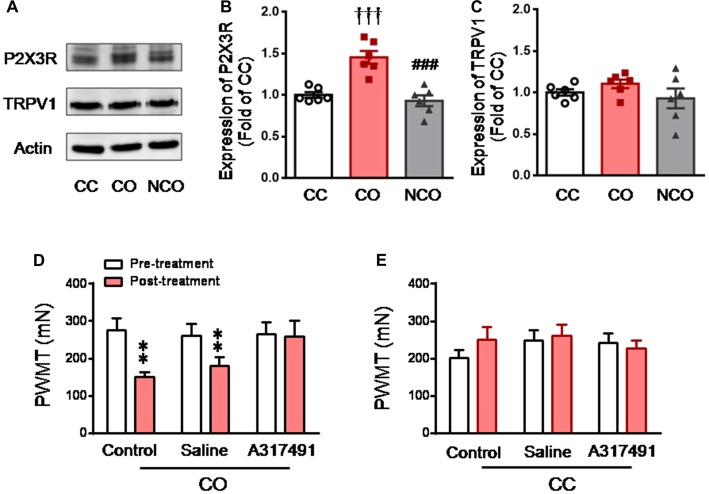
Enhanced P2X3 nociceptor expression in the DRG and blocking effect of P2X3 receptor antagonist on the empathic mechanical pain hypersensitivity in the CO rats.** (A–C)** showing representative immunoblotting bands and quantitative analysis of P2X3 and TRPV1 expressions in the L4-L5 DRG neurons in the CC, CO and NCO rats (sample *n* = 6 for each group, however, each sample contains DRGs obtained from at least 9 rats) following social interaction. The relative value density for bands of P2X3 and TRPV1 receptors in the CO and NCO rats was normalized to the value of P2X3 and TRPV1 receptors in the CC rats, respectively. **(D)** showing peripheral effects of P2X3 receptor antagonist (A317491) on development of the empathic mechanical pain hypersensitivity in the CO rats (saline, *n* = 9) and A317491, *n* = 16). **(E)** showing peripheral effects of P2X3 receptor antagonist (A317491) on baseline PWMT in the CC rats (A317491 and saline, *n* = 8 for each group). CC, cagemate control; CO, cagemate observer; DRG, dorsal root ganglia; NCO, non-cagemate observer; PWMT, paw withdraw mechanical threshold. ^†††^*P* < 0.001 CO vs. CC. ^###^*P* < 0.001 NCO vs. CO; **P < 0.01 vs. pre-treatment. Data are expressed as mean ± SEM.

After 30 min dyadic social interaction with a conspecific in pain, the expression level of DRG P2X3 derived from rats of the CO group was significantly increased (CO vs. CC or NCO, *P* < 0.001, *n* = 6 for each group), however, that from the NCO group was not changed relative to the CC (NCO vs. CC, *P* > 0.05; *n* = 6 for each group; Figures 3A,B). In sharp contrast, the expression level of TRPV1 was not changed by social interaction with a conspecific in pain in either the CO or NCO rats, relative to the CC (CO vs. CC or NCO vs. CC, *P* > 0.05, *n* = 6 for each group; Figures 3A,C).

To see whether peripheral P2X3 receptor is involved in mediation of empathic mechanical hyperalgesia, s.c. administration of A317491, a selective P2X3 receptor antagonist, was conducted randomly into the left or right hindpaw of both the CC and CO rats 15 min prior to dyadic social interaction. The results showed that pre-antagonism against P2X3 receptors with A317491 (0.2 mg/10 μl) prevented the empathic mechanical hyperalgesia from occurring in the injected limb of the CO rats (Post-treatment vs. Pre-treatment, *P* > 0.05, *n* = 16; Figure 3D), while the empathic mechanical hyperalgesia remained unchanged in the non-injected hindlimb (data not shown). As seen in the naïve CO rats (control), pre-saline administration in the CO rats failed to prevent the empathic mechanical hyperalgesia from occurring (Post-treatment vs. Pre-treatment, *P* < 0.01, *n* = 9; Figure 3D). Pre-administration of A317491 did not alter baseline PWMT in the CC rats relative to saline (Post-treatment vs. Pre-treatment, *P* > 0.05, *n* = 8 for both drug and saline; Figure 3E).

### Increased Neuronal Activity Was seen in the LC of CO but Not NCO Rats Following Social Interaction with a Conspecific in Pain

**Figure 4:**
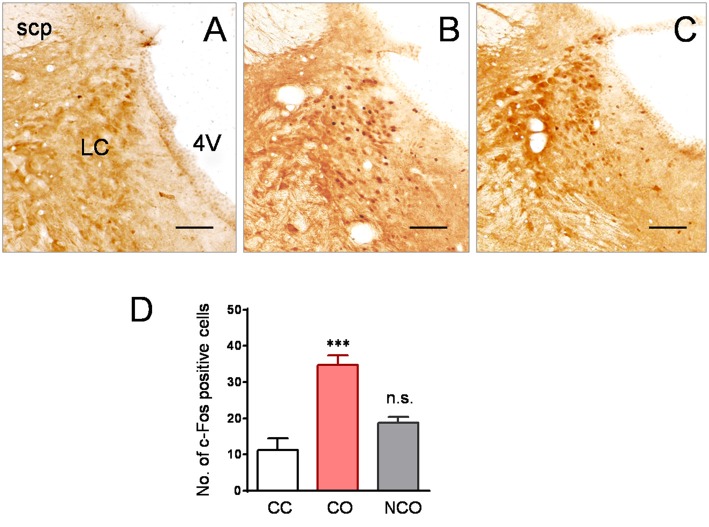
Specifically increased neuronal activity labeled by c-Fos protein was seen in the LC of the CO rats relative to the CC and NCO after 30 min dyadic social interaction with a conspecific in pain.** (A–C)** showing immunohistochemical images of c-Fos positive cells in the LC of the CC **(A)**, CO **(B)** and NCO **(C)** rats, following 30 min of social interaction. **(D)** sBlocked by Chemical Sympathectomyhowing averaged numbers of c-Fos positive cells in the LC of the CC, CO and NCO rats. 4V, fourth ventricle; CC, cagemate control; CO, cagemate observer; NCO, non-cagemate observer; LC, locus coeruleus; scp, superior cerebellar peduncle. Scale bar = 200 μm. ***P < 0.001 CO vs. CC; n.s., *P* > 0.05 NCO vs. CC. Data are expressed as mean ± SEM.

To see whether the LC is involved in mediation of empathic mechanical hyperalgesia in the CO rats after witnessing a familiar in pain, neuronal activity was examined using c-Fos immunoreactive labeling. As shown in Figures 4A,B, following 30 min social interaction with a familiar in pain, c-Fos positive cells in the LC of the CO rats were remarkably increased, relative to the CC rats (*P* < 0.001; *n* = 3 animals for each group). However, c-Fos positive cells in the LC of the NCO rats were not significantly increased relative to the CC rats (*P* > 0.05; *n* = 3 for the NCO) following 30 min social interaction with an unfamiliar in pain (Figures 4C,D).

### Evidence for the Essential Role of LC-NE System in P2X3 Receptor Over-Expression and Development of Empathic Mechanical Hyperalgesia in the CO Rats

To study the roles of the LC-NE system in over-expression of P2X3 receptor and development of empathic mechanical hyperalgesia that was specifically identified in the CO rats, the LC-NE neurons were destroyed by chemical neurotoxin DSP-4.

**Figure 5:**
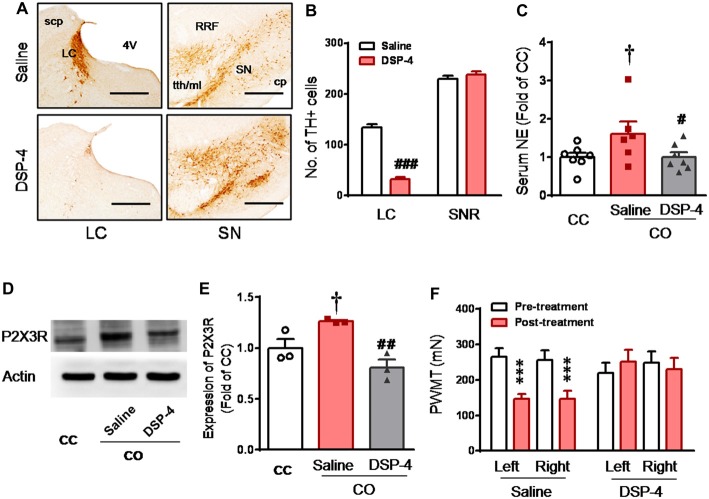
Selective destruction of the LC noradrenergic neurons/terminals by systemic neurotoxin DSP-4 and its effects on the level of circulating NE, expression of DRG P2X3 and development of the empathic mechanical pain hypersensitivity in the CO rats.** (A,B)** Immunohistochemical images and averaged number of tyrosine hydroxylase (TH)-labeled neurons in the LC and the substantia nigra (SN) in rats treated with DSP-4 and saline (*n* = 3 for each group). **(C)** showing serum NE levels in the CC (*n* = 7) and CO rats with pre-treatment of i.p. saline (*n* = 6) or DSP-4 (*n* = 7), following 30 min social interaction. **(D,E)** showing representative immunoblotting bands and quantitative analysis of P2X3 expression in the L4-L5 DRG neurons of the CC and CO rats with pre-treatment of i.p. saline or DSP-4 (sample *n* = 3 for each group, however, each sample contains DRGs obtained from at least 9 rats), following social interaction. The relative value density for bands of P2X3 receptors of CO rats was normalized to that in CC rats. **(F)** showing effects of destruction of the LC noradrenergic neurons/terminals on development of the empathic mechanical pain hypersensitivity (*n* = 11 for both saline and DSP-4 group). CC, cagemate control; CO, cagemate observer; cp, cerebral peduncle; NE, norepinephrine; PWMT, paw withdraw mechanical threshold; RRF, retrorubral field; SN, the substantia nigra; TH, tyrosine hydroxylase; tth/ml, trigeminothalamic tract/medial lemniscus. ^#^*P* < 0.05, ^##^*P* < 0.01, ^###^*P* < 0.001 DSP-4 vs. saline for CO; ^†^*P* < 0.05 Saline CO vs. CC; ***P < 0.001 vs. pre-treatment. Scale bar for **(A)** 500 μm. Data are expressed as mean ± SEM.

DSP-4 is known to be able to selectively delete LC noradrenergic terminals through i.p. administration *in vivo* (Jaim-Etcheverry and Zieher, 1980; Ross and Stenfors, 2015). In the current study, following 15 days after administration of DSP-4 (50 mg/kg, i.p.), the TH-labeled noradrenergic neurons in the LC were reduced by about 76%, compared to rats with saline treatment (DSP-4 vs. Saline, *P* < 0.001, *n* = 3 for each group; left panel, Figures 5A,B). However, the DSP-4 treatment exerted no effects on dopaminergic neurons in the SN (right panel, Figures 5A,B). Central lesion of the LC noradrenergic neurons in the CO rats abolished both the elevation of serum NE (DSP-4 vs. Saline, *P* < 0.05; *n* = 7 for each group) and P2X3 receptor over-expression in the DRG (DSP-4 vs. Saline, *P* < 0.01, *n* = 3 for each group; Figures 5C–E). The empathic mechanical hyperalgesia disappeared completely in the CO rats with the LC lesioned by DSP-4 (*n* = 11), while the behavioral phenomenon remained unchanged following i.p. saline treatment (*n* = 11; Figure 5F).

### Suppression of Central Noradrenergic NE Release by Clonidine Blocked P2X3 Over-Expression and Occurrence of Empathic Mechanical Hyperalgesia in the CO Rats

**Figure 6:**
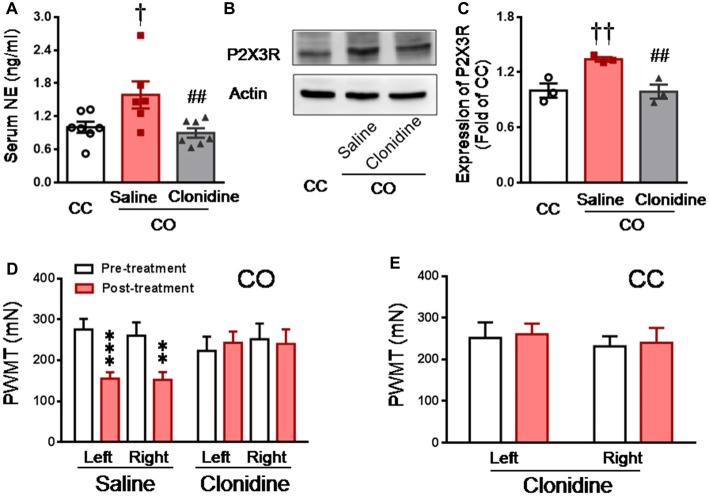
Inhibition of central NE release by systemic clonidine and its effects on expression of DRG P2X3 and development of empathic mechanical pain hypersensitivity in the CO rats.** (A)** showing serum NE levels in CC (*n* = 7) and CO rats with pre-treatment of i.p. saline (*n* = 6) or clonidine (*n* = 7), following 30 min social interaction. **(B,C)** showing representative immunoblotting bands and quantitative analysis of P2X3 expression in the L4-L5 DRG neurons of the CC and CO rats with pre-treatment of i.p. saline or clonidine (sample *n* = 3 for each group, however, each sample contains DRGs obtained from at least 9 rats), following social interaction. The relative value density for bands of P2X3 receptors of CO rats was normalized to that in CC rats.** (D)** showing inhibitory effects of central NE release by systemic clonidine on the development of empathic mechanical pain hypersensitivity (saline, *n* = 11 and clonidine, *n* = 7). **(E)** showing effects of i.p. clonidine (*n* = 7) on baseline PWMT in the CC rats. CC, cagemate control; CO, cagemate observer; NE, norepinephrine; PWMT, paw withdraw mechanical threshold. ^†^*P* < 0.05, ^††^*P* < 0.01 Saline CO vs. CC; ^##^*P* < 0.01 Clonidine vs. Saline for CO; **P < 0.01, ***P < 0.001 vs. pre-treatment. Data are expressed as mean ± SEM.

Because clonidine is well known for its suppressing action on pre-synaptic NE release through α2-adrenoceptor agonism (Jørum, 1988; Berridge and Waterhouse, 2003), the effect of clonidine was investigated in the CO rats after witnessing a familiar in pain. As shown in Figure 6A, the level of serum NE was significantly increased in the CO rats receiving i.p. pre-administration of saline (*n* = 6) relative to the CC rats (Saline CO vs. CC, *P* < 0.05, *n* = 6 or 7), however, serum NE was not significantly increased in the CO rats receiving i.p. pre-treatment of clonidine, relative to the CC (Clonidine CO vs. CC, *P* > 0.05, *n* = 7 for each group). The level of serum NE was significantly lowered in the CO rats with clonidine compared to those with saline (Clonidine vs. Saline, *P* < 0.01, *n* = 6 or 7).

Similarly, Western blot results showed that the expression level of P2X3 in the DRG was significantly increased in the CO rats receiving i.p. saline, relative to the CC rats (Saline CO vs. CC, *P* < 0.01, *n* = 3 for each group), however, P2X3 expression was not changed in the CO rats receiving i.p. clonidine, relative to the CC (Clonidine CO vs. CC, *P* > 0.05, *n* = 3 for each group; Figures 6B,C). The level of P2X3 expression was significantly lowered in the CO rats with clonidine compared to those with saline (Clonidine vs. Saline, *P* < 0.01, *n* = 3 for each group; Figures 6B,C).

In behavioral assays, the empathic mechanical hyperalgesia could be identified in the CO rats receiving i.p. saline (post-treatment vs. pre-treatment, *P* < 0.001, *n* = 11 for both limbs), but not in those animals receiving i.p. clonidine (Post-treatment vs. pre-treatment, *P* > 0.05, *n* = 7 for both limbs; Figure 6D). Treatment of clonidine in the CC rats did not change the baseline PWMT (*n* = 7, for both limbs; Figure 6E).

**Figure 7:**
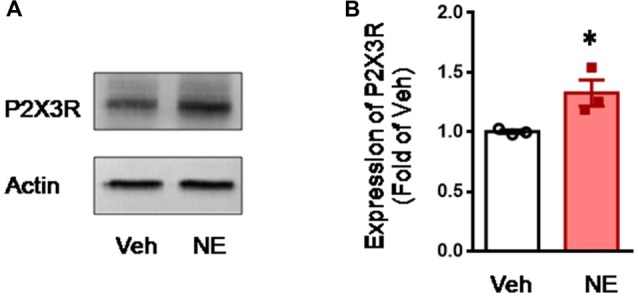
Incubation of acutely dissociated DRG neurons with NE upregulates the expression of P2X3 receptor. **(A,B)** showing representative immunoblotting bands and quantitative analysis of the expression of P2X3 in acutely dissociated DRG neurons following incubation with NE. The relative value density for bands of P2X3 treated with NE was normalized to the value of P2X3 treated with vehicle group. NE, norepinephrine; Veh, vehicle. *P < 0.05 vs. Veh. Data are expressed as mean ± SEM.

To confirm the effects of NE on the expression of P2X3, direct incubation of acutely dissociated L4-L5 DRGs with NE was performed. Similar to the previous report (Tan et al., 2011), incubation with NE could promote expression of P2X3 receptors in the DRGs relative to the vehicle control (NE vs. Veh, *P* < 0.05, *n* = 3 for each group; Figure 7).

### Behavioral Empathic Mechanical Hyperalgesia in the CO Rats Can be Blocked by Chemical Sympathectomy

**Figure 8:**
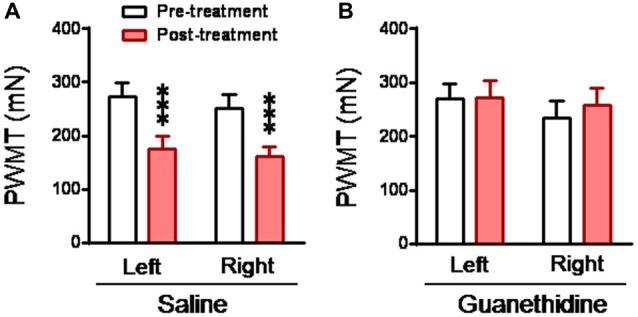
Abolishment of empathic mechanical pain hypersensitivity by chemical sympathectomy with guanethidine in the CO rats. **(A,B)** showing changes in PWMT in the CO rats, which received s.c. saline (*n* = 11) or guanethidine (*n* = 11; 50 mg/kg/day for 3 days), before and after social interaction with a cagemate in pain. ***P < 0.001 vs. pre-treatment. Data are expressed as mean ± SEM.

To see whether the mediating role of the LC-NE system in the development of empathic mechanical hyperalgesia is through sympathetic ganglia, sympathetic trunk was chemically destroyed by systemic guanethidine. The CO rats with sympathectomy by systemic guanethidine (*n* = 11) did not show empathic mechanical hyperalgesia at all, while those with intact sympathetic structure (saline treatment, *n* = 11) did show empathic mechanical hyperalgesia after dyadic social interaction with a familiar in pain (Figure 8).

### Empathic Mechanical Hyperalgesia Can be Blocked by α1, but Not α2 or β, Adrenoceptor Antagonist in the Periphery

**Figure 9:**
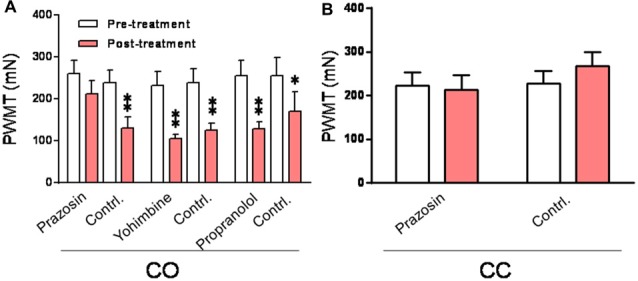
Peripheral effects of α1, α2 and β adrenoceptor antagonists on development of the empathic mechanical pain hypersensitivity in the CO rats.** (A)** showing changes in PWMT in the CO rats with pre-administration of prazosin (*n* = 12), yohimbine (*n* = 7) and propranolol (*n* = 7), respectively, into one hand paw (drug side). **(B)** showing effect of prazosin on the baseline PWMT in the CC rats (*n* = 9). Contrl., contralateral side to the drug injection; PWMT, paw withdraw mechanical threshold. *P < 0.05, **P < 0.01 vs. pre-treatment. Data are expressed as mean ± SEM.

It is known that the sympatho-postganglionic NE innervations have little effect on pain under physiological condition, however, NE can enhance pain or hyperalgesia under pathological conditions such as occurrence of peripheral inflammation and nerve injury (Jones, 1991; Pertovaara, 2006). Here we tested the peripheral effects of α1, α2 and β adrenoceptor antagonists on the development of empathic mechanical hyperalgesia. As shown in Figure 9A, the empathic mechanical hyperalgesia of the injection side was blocked by s.c. pre-administration of prazosin into unilateral hindpaw (left or right in random order) 15 min prior to the dyadic social interaction with a familiar in pain (Post-treatment vs. Pre-treatment for prazosin, *P* > 0.05), whereas, that of non-injection side was not affected (Post-treatment vs. Pre-treatment for contrl., *P* < 0.01; *n* = 12 for each group). In sharp contrast, however, the same treatment of either yohimbine (*n* = 7) or propranolol (*n* = 7) did not have any effects on the empathic mechanical hyperalgesia (Post-treatment vs. Pre-treatment for yohimbine or propranolol of both side, *P* < 0.01, *n* = 7 for each group). Subcutaneous administration of prazosin similarly in the CC rats exerted no effects on the baseline PWMT of both the injected and non-injected hindpaws (Figure 9B).

## Discussion

### Does the mPFC-LC/NE Neural Circuit Mediate Empathy for Pain?

Empathy is an evolutionary behavior of social animals and humans associated with prosocial reciprocity, altruism and morality by the ability to feel, recognize, understand and share the emotional states of others (Preston and de Waal, 2002; de Waal, 2008; Gonzalez-Liencres et al., 2013; Panksepp and Panksepp, 2013; Decety et al., 2016; Sivaselvachandran et al., 2016; Chen, 2017). Due to paucity of animal experimental research, so far, the molecular and neural mechanisms underlying development of empathy are largely unknown. In a rat model of empathy for pain, chemical lesions of the bilateral mPFC, a cortical structure that includes the prelimbic cortex (PrLC), infralimbic cortex (ILC) and the ACC, have been demonstrated to abolish development of the empathic mechanical hyperalgesia in the CO rats, however, bilateral amygdala and entorhinal cortices lesions failed to affect this empathic response to a familiar conspecific in pain (Li et al., 2014). In a mouse model of empathy for fear, the medial thalamus-ACC pathway has been shown to be critical in mediation of observational fear learning with Cav1.2 voltage-gated calcium channel of the ACC being involved (Jeon et al., 2010). In another rat model of empathy for fear, social interaction for 10 min with a recently fear conditioned cagemate in the home cage resulted in increase in c-Fos expression in the mPFC (PrLC and ILC) and amygdala (Knapska et al., 2006; Mikosz et al., 2015). Collectively, the mPFC is likely to play a critical role in mediation of both empathy for pain and empathy for fear in rodents at the cortical level (Li et al., 2014; for review, see Chen, 2017).

In the current study, we provided another line of evidence showing that dyadic social interaction with a familiar conspecific in pain for 30 min can activate the LC-NE system, resulting in more release of NE into the circulation through driving the SAM outflow. The activation of the LC-NE system could be mediated by top-down modulation from the mPFC, because there is plenty of evidence showing tight bidirectional synaptic connections and functions between the mPFC and the LC noradrenergic neurons (Arnsten and Goldman-Rakic, 1984; Sara and Segal, 1991; Jodo et al., 1998; Aston-Jones et al., 1999; Berridge and Waterhouse, 2003; Bouret and Sara, 2005; Sara, 2009; Sara and Bouret, 2012). The activated descending LC noradrenergic neurons drive the cholinergic sympathetic preganglionic neurons in the spinal intermediolateral (IML) column. The IML cholinergic sympathetic preganglionic neuronal outflow further activates chromaffin cells in the adrenal medulla, on one hand, and/or activates sympathetic postganglionic noradrenergic neurons in the sympathetic ganglia, on the other hand, consequently leading to elevation of the circulating NE and/or release of NE in the skin through sympathetic innervations. This presumption can be supported by morphological evidence showing dense descending noradrenergic nerve fiber terminals (boutons) projected from the A6 (LC) and A5 areas of the pons to the spinal IML column, and getting close proximity to the cholinergic sympathetic preganglionic neurons (Bruinstroop et al., 2012). A line of functional evidence is also supportive since it has been shown that activation of the central noradrenergic neurons in the A5 area resulted in increase in sympathetic nerve discharges in rats (Huangfu et al., 1991). Besides the role of P2X3 receptors in the peripheral mechanisms of the empathic mechanical hyperalgesia found in this study, α1 subtype of adrenoceptors is also likely to be involved because local pre-administration of prazosin, an α1 adrenoceptor antagonist, through s.c. route of delivery, could prevent the empathic mechanical hyperalgesia from occurring, however, the same treatment of either yohimbine or propranolol, α2 or β adrenoceptor antagonist, did not have such effect. Taken together, the empathic mechanical hyperalgesia is likely to be mediated by a neural circuit that involves both the SAM axis NE release and the sympatho-postganglionic NE release driven by the mPFC-LC/NE system.

As for the neural mechanisms underlying activation of the LC/NE system by empathic response to pain of others, so far little is known. In a mouse model, it has been demonstrated that blocking the visual sensory input could abolish empathy-associated social pain contagion, while destruction of olfactory and auditory sensory input did not have such effect (Langford et al., 2006), implicating an important role of visual sensation in the development of empathy for pain. Recently, it was shown that witnessing a conspecific displaying spontaneous itch from the neighbor transparent homecage or the behaviors displayed by a video on computer screen could activate neurons in the suprachiasmatic nucleus (SCN) of the hypothalamus and elicit contagion of itch in naïve observer mice (Yu et al., 2017). The social transfer of itch through visual information was further demonstrated to be mediated by a neural circuit in the SCN through gastrin-releasing peptide (GRP)-GRP receptor signaling (Yu et al., 2017). Whether this non-image forming pathway mediated visual information to the SCN (Berson et al., 2002; Hattar et al., 2002) is involved in social transfer of pain (empathy for pain) is unknown and is worthy of being further studied. The LC is known to be highly associated with vigilance, attention, and cognition that can be evoked by various sources of sensory salience (Sara and Segal, 1991; Aston-Jones et al., 1999; Berridge and Waterhouse, 2003; Bouret and Sara, 2005; Sara, 2009; Sara and Bouret, 2012). Neuroimaging study in human subjects has shown that during social interaction with a familiar in pain may increase the perceived intensity of pain sensation through visual sensory pathway (Villemure et al., 2003; Villemure and Bushnell, 2009), resulting in activation of the ACC and insula but not the somatosensory cortex (Singer et al., 2004; Lamm et al., 2011). How visual information is transmitted to activate the mPFC and LC/NE system is of particular interest and requires to be further studied at the neural circuit level using techniques of optogenetics.

### Mediation of Empathy for Pain by the LC/NE-SAM Axis, but Not by the HPA Axis

The most interesting finding of the present study is that social interaction with a familiar or an unfamiliar conspecific in pain had different responses of the SAM and HPA axis. Specifically, social interaction with a familiar conspecific in pain resulted in hyperactivity of LC/NE-SAM system without any alteration in the HPA activity, leading to up-regulation of P2X3 receptor in the DRG and reduced mechanical pain threshold (a biomarker of empathy for pain). However, social interaction with an unfamiliar conspecific in pain resulted in hyperactivity of both SAM and HPA axis but without over-expression of P2X3 receptor in the DRG and behaviors associated with empathy for pain (lowered mechanical pain threshold). Although the increased circulating level of NE was seen in the NCO rats after social interaction with an unfamiliar in pain, it is not likely to be caused by activation of the LC-NE system, because c-Fos-labeled neuronal activity in the LC was not increased in the NCO rats. Nonetheless, the increased NE level seen in the NCO rats could be explained as caused by social stress associated with the increased HPA activity and other changes. Social stress often occurs in a strange environment or among unfamiliar strangers with activation of the HPA axis, resulting in elevation of circulating CORT in both humans and rodents (Chrousos, 2009). Actually, it was found that both pharmacological blockade of the synthesis of CORT in the adrenal cortex and antagonizing the GC and MC receptors with antagonists enabled emotional contagion of pain in both human participants and mice among strangers who would have failed to do so if the HPA axis hyperactivity had existed (Martin et al., 2015). Similarly, priming social game among strangers (e.g., cooperative experience to play rock and roll music together) could reduce both the elevated circulating CORT and social stress, enabling emotional contagion of pain among dyadic stranger participants as well (Martin et al., 2015). It was also shown in the current study that the rise in the circulating CORT was only measurable in the NCO rats 10 min after social interaction with an unfamiliar conspecific in pain, however, no change was seen in either the CC or the CO rats after social interaction with a familiar without pain or a familiar in pain (Li et al., 2014). Moreover, in sharp contrast, social interaction with an unfamiliar conspecific in pain was shown to result in social stress-induced analgesia, but not hyperalgesia, in the NCO mice (Langford et al., 2011). Taken together, it suggests that social contact with a familiar conspecific in pain results in social approach (empathy-associated hyperalgesia) driven by activation of the mPFC-LC/NE-SAM axis, however, social contact with an unfamiliar conspecific in pain results in social stress (stress-associated analgesia) initially co-driven by the HPA and SAM hyperactivity and then driven by the SAM axis mediated by a non-LC/NE input. The involvement of the LC/NE-SAM system in empathy for pain can be largely supported by human studies showing enhancing effects of the NE (noradrenaline) on empathy-based prosocial behavior, intergroup relations and moral decisions (Terbeck et al., 2016).

Stress is well known to have dual modulatory effects on pain, referred to as stress-induced analgesia (Butler and Finn, 2009) and stress-induced hyperalgesia (Imbe et al., 2006; Jennings et al., 2014; Olango and Finn, 2014). It has been suggested that less intensive stressors can evoke stress-induced hyperalgesia, whereas more severe stressors can lead to stress-induced analgesia (Williams and Rhudy, 2007; Langford et al., 2011; Olango and Finn, 2014). However, unlike stress response to various stressors, the empathic response prefers to use the mPFC-LC/NE-SAM system rather than the HPA axis. This is probably due to the biological processes of the familiarity (social bond) among people or among animals (also cross-species such as human-pet relationship) that relieve stress through inhibition of the HPA axis by oxytocin, a prosocial neuropeptide released from the hypothalamus (Neumann et al., 2000; Heinrichs et al., 2003; Engelmann et al., 2004; Lukas et al., 2011; Chen, 2017). This presumption can be supported by many studies demonstrating that intranasal administration of oxytocin has anti-HPA effect and relieves social stress in both humans and rodents (Neumann et al., 2000; Lukas et al., 2011; Chen, 2017). Intranasal oxytocin has also been shown to enhance empathy for pain and activity in the ACC and anterior insula where empathy is processed (Singer et al., 2008). As shown in our and other’s previous reports, the social transfer of pain through empathic responses is specifically dependent upon establishment of familiarity that requires co-house of the rodents (cagemates) for at least 2–3 weeks regardless of whether they were siblings or not (Langford et al., 2006; Li et al., 2014). The establishment of social bond (familiarity) has also been shown to be an essential condition for a rat to release a distressing cagemate from a restrainer (Ben-Ami Bartal et al., 2011), a behavior that is explained to be motivated by prosocialism and altruism. It would be very interesting to see whether oxytocin has a role in mediating empathy for pain in rodents (Chen, 2017).

### Selective Up-Regulation of Primary P2X3 Nociceptor by LC/NE-SAM Axis Activation Serves as Molecular Basis for Development of Empathic Mechanical Hyperalgesia

In the current study, we also provided another line of new evidence showing that the elevated circulating NE driven by the LC/NE-SAM system selectively up-regulates the expression of P2X3 receptors, but not thermal nociceptor TRPV1, in the DRG that serves as peripheral molecular basis for development of the empathic mechanical hyperalgesia identified in the CO rats.

It is interesting to note that the nociceptive responses mediated by P2X3 receptors in the periphery can be enhanced by noradrenergic innervations (Maruo et al., 2006; Meisner et al., 2007). Moreover, direct incubation of the cultured DRG neurons with NE has also been shown to up-regulate the expression of P2X3 receptors by both a previous report (Tan et al., 2011) and our current study. This suggests existence of an enhancing effect of the LC/NE-SAM system on the peripheral nociception mediated by P2X3 nociceptor molecule. In the current study, we further showed that the over-expression of P2X3 proteins in the DRG was selectively identified in rats with the familiarity-based empathy for pain and dependent upon the elevated circulating NE caused by the LC/NE-SAM axis activation evidenced by a series of experiments. However, thermal nociceptor molecule TRPV1 was not changed under the same condition. This can well explain why only mechanical hyperalgesia occurs in rats with empathy for pain. It has long been known that the LC/NE system can modulate pain through both supraspinal and spinal actions (Jørum, 1988; Proudfit, 1988; Jones, 1991; Pertovaara, 2006). Traditionally the descending LC/NE system to the dorsal horn of the spinal cord has been demonstrated to produce anti-nociceptive action together with serotonin (Proudfit, 1988; Jones, 1991; Pertovaara, 2006). However, in the periphery, the actions of NE are complex. Under physiological state, NE does not evoke pain after s.c. administration, nor does it affect primary nociceptive neuronal activity when being injected into the receptive field of the relevant neuronal unit (Jones, 1991; Pertovaara, 2006). However, under pathological conditions induced by tissue or nerve injury, administration of NE into the peripheral receptive field activates its nociceptor cell to discharge and produces or enhances pain (hyperalgesia; Jørum, 1988; Pertovaara, 2006). Moreover, it has been shown that the DRG primary cells can be innervated by sympathetic sprouting basket under peripheral nerve injury and this phenomenon is believed to be associated with sympathetically-maintained pain observed in chronic regional pain syndrome. Herewith, in supporting the enhancing effect of NE on P2X3 receptors in the periphery, we further revealed that endogenous NE in the skin was essential to the development of the empathic mechanical hyperalgesia because pre-blockade of α1 adrenoceptor with prazosin could prevent its occurrence. The existence of α1A, 1B and 1D subtypes of adrenoceptors in the DRG is strongly supportive for this action (Pertovaara, 2006), although the intracellular signaling pathway for the functional link between NE-α1 adrenoceptor and ATP-P2X3 receptor in the development of the empathic mechanical hyperalgesia is yet known. Together, here we added one more line of evidence showing that, on one hand, P2X3 nociceptor of the primary sensory neurons can be up-regulated by high level of circulating NE maintained by empathy-induced LC/NE-SAM axis activation, while on the other hand, the P2X3 receptors anterogradely transported and trafficked from the cell body to the peripheral nerve endings would be further regulated via activation of α1 adrenoceptor by endogenous NE released from the sympathetic postganglionic neurons, which together serve as a molecular basis for development of familiarity-based empathic mechanical hyperalgesia.

### Limitations of the Current Study

In the current study, it was paradoxical to note that the circulating NE was increased in the NCO rats after social interaction with an unfamiliar conspecific in pain, however, the expression level of P2X3 receptor in the DRG was not changed (Figures 2, 3). However, in the CO rats the increased expression of P2X3 receptor in the DRG was strongly associated with the elevated circulating NE after social interaction with a familiar conspecific in pain. We did not carry out the LC lesion and synaptic release inhibition experiments in the NCO rats because, unlike that in the CO rats, the neuronal activity in the LC was not significantly increased following social interaction with an unfamiliar conspecific in pain (Figure 4). Moreover, as discussed above, social stress occurred in the NCO rats in which both the SAM axis and HPA axis were hyperactive, while social approach shown as empathy for pain occurred in the CO rats in which only the LC/NE-SAM axis was hyperactive. These results suggest that the underlying mechanisms of social stress observed in the NCO rats are completely different from those of empathy for pain observed in the CO rats. Under such circumstance, other factors beyond the LC/NE-SAM system should be taken into account, for example, the functional balance between hypothalamic-neurohypophyseal system (such as oxytocin and vasopressin) and the HPA axis (such as CORT) under social stress and empathy requires to be further examined in the near future.

## Conclusion

In the current study, we found that the LC/NE-SAM system was involved in the mediation of empathy for pain in the CO rats through up-regulating P2X3 receptors in the DRG by elevating circulating NE. Meanwhile, in the periphery, the sympatho-postganglionic source of NE might enhance the action of P2X3 receptor through acting on α1 adrenoceptor. These together serve as molecular and peripheral neural basis for the development of empathic mechanical hyperalgesia in rats.

## Author Contributions

ZL and JC conceived the study, ZL, Y-FL, YY, C-LL and YW performed the experiments and carried out data analysis. ZL and JC drafted the manuscript.

## Conflict of Interest Statement

The authors declare that the research was conducted in the absence of any commercial or financial relationships that could be construed as a potential conflict of interest.
